# Early Spatial Frequency Processing of Natural Images: An ERP Study

**DOI:** 10.1371/journal.pone.0065103

**Published:** 2013-05-31

**Authors:** Andrea De Cesarei, Serena Mastria, Maurizio Codispoti

**Affiliations:** Department of Psychology, University of Bologna, Bologna, Italy; University of Rome, Italy

## Abstract

The present study examined the role of spatial stimulus frequencies in the early visual processing of natural scenes. The content of initially degraded (low- or high-pass filtered) pictures was progressively revealed in a sequence of steps by adding high or low spatial frequencies. Event Related Potentials (ERPs) were used to track the early stages of visual processing. Picture degradation modulated the topography of the P1, with an occipital midline distribution for the most degraded pictures, which became progressively more laterally distributed as pictures became more complete. Picture degradation also modulated the amplitude of the P2. For both low-passed and high-passed scenes, a linear relationship between the spectral power and the amplitude of the P1 and P2 was observed. These results are likely to reflect the progressive engagement of the lateral occipital complex as the amount of information in both the low and high portions of the frequency spectrum increased.

## Introduction

**Figure 1:**
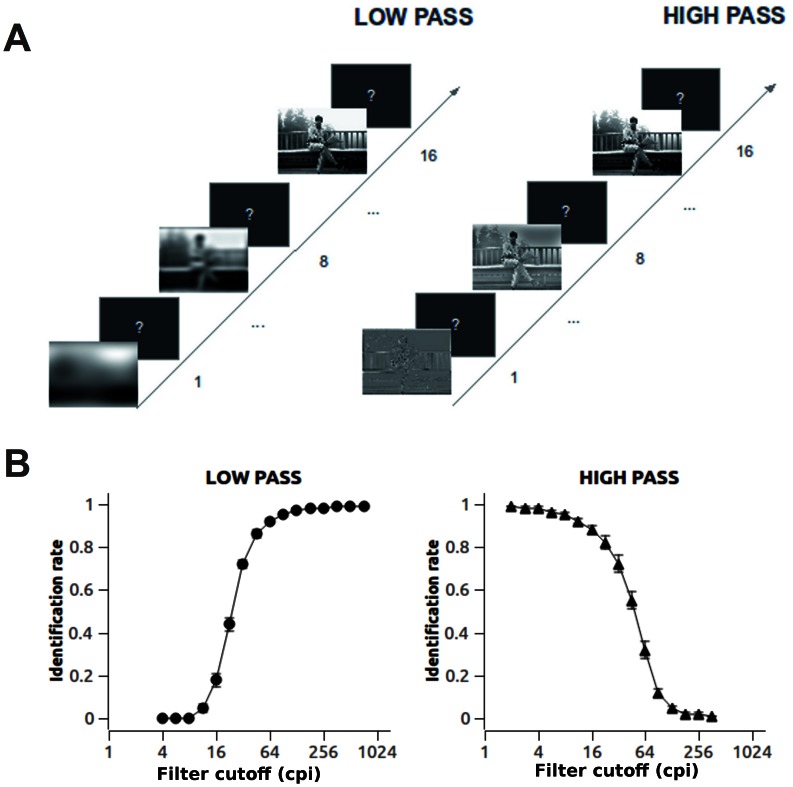
Outline of the experimental procedure and of the stimulus identification performance. A. Experimental procedure. In each trial, an initial low-pass or high-passed version of a natural scene was presented. In the following steps, the severity of the spatial filtering was loosened to include a wider range of frequencies, until most of the original image was presented at step 16. B. Identification performance in the low-pass (left) and high-pass (right) condition. As high spatial frequencies were added in the low-pass condition (left), or low spatial frequencies were added in the high-pass condition (right), identification rate increased.

The visual system continuously translates visual input into coherent high-level representations. This remarkable efficiency results from the activity of neural pathways and structures, which analyze visual input through parallel and hierarchical processing. These processes analyze features of increasing complexity, eventually leading to complex perceptual phenomena such as grouping, categorization and identification [1], [2]. Visual input contains edges and contrast changes, and this information can be described in terms of spatial frequencies [3]. The spatial frequency spectrum correlates with the ratio between the total area of visual input and the area of an element. Low spatial frequencies describe constrast changes which happen across large areas of an image while high spatial frequencies refer to contrast changes which happen across small portions of a scene (Figure 1). In the early visual processing stages, image contrast is analyzed in several spatial frequency bands and contributes to the identification of the visual input [4]–[6].

Several studies have investigated which spatial frequency information is analyzed at distinct processing stages. Event-Related Potentials (ERPs) have revealed themselves to be a useful tool in such research, as they allow one to observe electrocortical activity with a fine-grained temporal resolution. Using simple stimuli such as gratings of checkerboards, it has been shown that the early C1 component (which has been alternatively labeled N1 in the visual perception literature [7]) is sensitive to the amount of contrast in the high spatial frequency range, while the P1 and P2 amplitudes are enhanced for stimuli with high contrast in the low spatial frequency range [7]–[9].

A number of studies have focused on object identification, examining the neural mechanisms which underlie the recognition of degraded figures [10], [11]. In a typical paradigm, pictures or objects are initially presented in a degraded form, and information is gradually added until participants are able to identify the stimulus [12], [13]. As far as ERPs are concerned, a more pronounced P2 was observed for intact compared to fragmented drawings, suggesting that P2 is related to object identification [11].

## The Research Problem

Most previous ERP studies have investigated the use of visual information using gratings or checkerboards as stimuli. Although these types of stimuli are easy to generate and control for a number of features, they are remarkably different from the natural scenes we deal with most of the time. As such, the utility of studying the response to gratings and checkerboards to understand the functioning of the visual system has been questioned [14], [15]. Studies using simple drawings represent an intermediate step between gratings and natural scenes [11]. However, less is known regarding the use of spatial frequency information for natural scene identification.

The identification of natural scenes is strictly related to the amount of information present. The amount of sensory information in typical scenes correlates with the power distribution within the frequency spectrum of an image [16]. The spectral power indicates the amount of contrast within a spatial frequency range. For instance, pictures which have a large spectral power in the low frequency range will have coarse regions which are highly contrasted. On the other hand, scenes with a low spectral power in the low frequency range will be almost uniform in their coarse components. Most relevantly, degraded pictures will have a lower spectral contrast power compared to intact scenes. It has been shown that activation of early visual areas (V1 and extrastriate) depend on the perceived image contrast [17], and it is possible that the increase in amplitude of the occipital P2 that was observed in previous studies depends on the increasing image contrast and spectral power.

The present study examined the use of spatial frequency information in the identification of natural scenes, as indexed by early ERPs. A filtering approach was used to disentangle the role of low and high spatial frequencies. Natural scenes were presented either in their low or high spatial frequency components. Within each type of filter, the amount of spatial frequencies was varied in a number of steps, and pictures varied from extremely degraded to intact. Spectral power was computed at each degradation step. This design allowed us to observe the modulation of early ERPs, as information was added and identification proceeded, based on the high or low spatial frequency information.

A general prediction, which can be expected for all ERP components, is that if a component is exclusively linked to either high-or low- pass information, then it will only be observed when that type of information is presented. Alternatively, if any type of spatial frequency information (i.e., either low or high) contributes to the processing stages reflected in an ERP component, then similar ERPs should be observed for stimuli composed of low and high spatial frequencies.

Moreover, the relationship between spectral power of the scene and electrocortical activity was quantified. If the activation of a visual area is related to the spectral power of an image, then a similar activity should be observed when pictures of similar spectral power are compared, regardless of the type of spatial frequency (low or high) displayed.

## Materials and Methods

### Ethics Statement

The study was approved by the Ethical Committee of the Department of Psychology at the University of Bologna, and a written informed consent was obtained from all participants.

### Participants

A total of 35 participants (18 females, age *M = *25.26, *SD = *4.79) took part in the study. Vision was normal or corrected to normal. Participants had no previous experience with the materials used in this experiment. In a previous study, data from the present dataset were analyzed in a later time interval to investigate the role of spatial frequencies and identification on a well-established ERP component of emotional modulation, namely the Late Positive Potential (LPP) [18].

### Stimuli and Equipment

A total of 60 grayscale images were selected for the testing phase, portraying people in neutral, pleasant or unpleasant contexts. Five additional pictures were used for a training phase. In a preliminary analysis, the same pattern of results was observed for the neutral category and for the whole picture set. To improve the generalizability of the present results and to increase the statistical power, results from the whole picture set are reported. The pictures were balanced for contrast and brightness (0.6 and 0.1 on a 0–1 linear scale) and subtended a visual angle of 15 (horizontal)×11 (vertical) degrees, measured from edge to edge of the image. The pictures were presented on a 21′′ CRT monitor which was 1.5 m away from the observer.

### Filter Parameters

From each original picture, 32 different versions were created using an in-house-developed Matlab script, by applying a low- or high-pass spatial frequency filter which varied in the cutoff level. For the low-pass filter, the cutoffs ranged from 4 to 724 cpi (4, 6, 8, 11, 16, 23, 32, 45, 64, 91, 128, 181, 256, 362, 512, 724), while for the high-pass filter they ranged from 362 to 2 cpi (362, 256, 181, 128, 91, 64, 45, 32, 23, 16, 11, 8, 6, 4, 3, 2). These filters were selected based on pilot data, which are not reported here.

The low-pass filter passed all spatial frequencies lower than the 1/3·cutoff, and eliminated all spatial frequencies equal to or higher than the cutoff value, with a parabolic slope [18]–[20]. The high-pass filter was identical to the low-pass filter, mirror-imaged on the frequency axis, and it passed all spatial frequencies higher than the 1/3·cutoff; all spatial frequencies lower than the cutoff value were eliminated with a parabolic slope.

The spectral power of all versions of all images was calculated using a Fourier transform and then circularly averaged. To obtain a single estimate of the spectral power for each picture version, the spectral power was averaged across all spatial frequencies.

### Procedure

Each trial began with the most degraded (either low- or high-passed) version (see Figure 1). A 500 ms fixation cross at the center of the screen preceded the presentation of a picture, which remained visible for 1 s. After picture offset, participants were asked to respond whether they identified the gist of the image or not, using a response box. In order to specify instructions that did not rely preferentially on local or global details, participants were told that identification was sufficient when the context of a scene (e.g., “indoor scene with people”) was understood. In contrast, understanding the perceptual layout (“I can see the contrast with the horizon”) or reliance on fine-grained details (“I can see something that looks like a flower…”) was insufficient as identification. No instructions relative to response speed were given, and the procedure was halted until the participant responded. After a response was given, and following an additional 1 s delay, the same picture was presented in a less degraded (higher low-pass cutoff or lower high-pass cutoff) version. This procedure was repeated for all 16 versions of a picture. Then, participants were asked whether their first “yes” response was correct or not, based on whether the initial gist was confirmed by the following, less degraded picture versions. Then, the identification procedure for the next picture started. Each experimental session started with five practice trials, which were followed by 60 test trials. This identification procedure had already been used in previous research, showing a similar use of spatial frequency information by this and other procedures, such as asking participants to decide whether a verbal descriptor matched the presented picture [19].

For each participant, each picture was presented either in the low or in the high-pass spatial filter condition, but not both, to prevent previous identification of a low-passed picture from influencing identification of the high-passed version, or viceversa. Across participants, all pictures were presented with an equally frequent occurrence in the low and high-pass spatial filter version. The first five pictures served as practice, and were not analyzed.

### EEG Recording and Processing

EEG was recorded at a sampling rate of 256 Hz from 256 active sites using an ActiveTwo Biosemi system. An additional sensor was placed below the participant’s left eye, to allow for detection of blinks and eye movements. The EEG was referenced to an additional reference electrode located near Cz during recording. A hardware 5th order low-pass filter with a −3dB attenuation factor at 50 Hz was applied online. Off-line analysis was performed using Emegs [21], and included filtering (0.1 Hz high-pass and 40 Hz low-pass), removal of eye movement artifacts [22], artifact detection and sensor interpolation, averaging across trials and conversion to an average reference montage. A baseline correction based on the 100 ms prior to stimulus onset was performed.

ERPs were scored in the scalp regions where amplitude across all subjects and conditions was maximal. For all components, a peak measure of amplitude (most positive or negative peak in the time interval of interest) was used. The C1 was scored at midline occipital sensor sites (14–17, 19–23, 25–29; see Figure S1, left) as the most negative peak in the 50 to 90 ms interval from stimulus onset. The P1 and N1 were scored at lateral occipital sensor sites (8–11, 37–44, 48–49, 144–145, 156–159, 190–193, 201, 219–220, 223–225; see Figure S1, center), as the most positive peak in the 80–120 ms time interval (P1) and as the most negative peak in the 150–190 ms time interval (N1). Additionally, the P1 was also scored at midline occipital sites (12–15, 21–28, 202, 210–211; see Figure S1, center). The P2 was scored at occipitotemporal sensor sites (9–14, 21–28, 37–42, 158–159; see Figure S1, right) as the most positive peak in the 150 to 280 ms interval from stimulus onset. These data were analyzed with an ANOVA with factors Type of Filter (high vs. low pass) and Step (16 levels). In the case of the P1, where a topographic question was assessed, an additional factor, Sensor Group (lateral vs. midline), was added to the ANOVA design. Following significant main effects, post-hoc tests were conducted.

In order to assess the effects of spectral power and identification on ERPs, a regression approach was used. First, ERPs were averaged in the 32 conditions defined by the Step×Type of Filter design. Then, a stepwise multiple regression analysis was carried out, using ERP amplitude as a dependent variable and spectral power, identification rate and spatial frequency as predictors. Predictors were progressively added to the regression model using a forward selection procedure.

### Source Analysis

Standardized low-resolution brain electromagnetic tomography (sLORETA) software was used to compute the cortical three-dimensional distribution of current density for the grand averaged ERPs [23]. Computations were made in a realistic head model, using the MNI152 template [24], with the three-dimensional solution space restricted to cortical gray matter, as determined by the probabilistic Talairach atlas [25]. Sensors positions were mapped to the MNI template by projecting the electrodes’ positions in the MNI152 template based on their spherical coordinates. The intracerebral volume was partitioned into 6239 voxels at a 5 mm spatial resolution. Anatomical labels such as Brodmann areas are also reported using MNI space, with correction to Talairach space [26].

## Results

### Identification

Picture identification is reported in Figure 1. At the initial steps, no identification was achieved. When low or high spatial frequency information was added, identification rate increased with similar slopes for low- and high-passed pictures. No significant differences in the absolute slope of the best-fitting Weibull psychometric function were observed for low-pass compared to high-pass filtered pictures.

### C1

**Figure 2:**
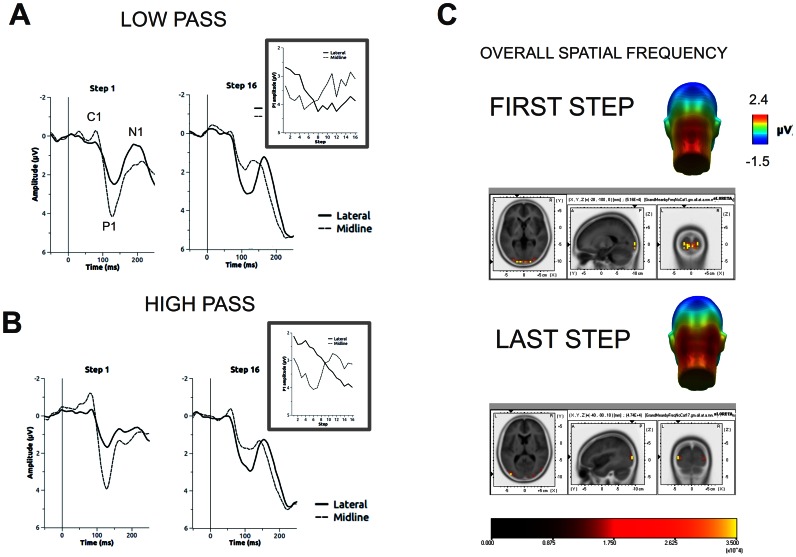
The effects of spatial frequencies on the early ERPs. A, B. For the first and last step of the procedure, waveforms are displayed in the three right-hand side panels. Waveforms represent the average across sensors in central and lateral sensor groups. The insets contain line plots displaying the effects of Step on the amplitude of the P1, at lateral and midline sites. In all plots, positive values are plotted downwards. C. Scalp topography and cortical localization of the P1 in the first and last step. In this representation, the low- and high-pass conditions were averaged together.

The effects of picture identification on the amplitude of the C1 are visible in Figure 2 (midline sites). The C1 was more negatively pronounced in the high-pass than in the low-pass condition, as seen in the main effect of Filter F(1, 34) = 14.94, p<.001, η^2^_p_ = .31. Additionally, the C1 was more negative at the initial steps than at the later steps of the identification procedure, as indicated by the main effect of Step F(15, 510) = 2.2, p<.05, η^2^_p_ = .06, linear contrast F(1, 34) = 10.32, p<.01, η^2^_p_ = .23. The interaction between Filter and Step approached standard significance, F(15, 510) = 1.68, p = .08, η^2^_p_ = .05. In the high pass condition, amplitude of the C1 was most negative at the first steps and decreased at later steps, F(15, 510) = 2.41, p<.01, η^2^_p_ = .07. In the low pass condition, no effects of Step were observed.

### P1

The results for the P1 component are shown in Figure 2. The topography of the P1 changed within the identification procedure. At the first steps, the P1 was more pronounced centrally than laterally, and this pattern was reversed at the final identification steps. This effect was similarly pronounced in the low-pass and in the high-pass condition. This effect was statistically supported by the significant interaction of Group (lateral vs. midline) and Step, F(15, 510) = 49.48, p<.001, η^2^_p_ = .59. At lateral sites the amplitude of the P1 increased with Step F(15, 510) = 16.62, p<.001, η^2^_p_ = .33, while at midline sites the opposite effect was observed, F(15, 510) = 8.1, p<.001, η^2^_p_ = .19.

The three-way interaction between Sensor Group, Cutoff and Type of Filter was not significant. However, a significant interaction between Sensor Group and Type of Filter indicated that in the high pass condition a less pronounced P1 amplitude was observed at lateral than at midline sites, while no effect of Sensor Group was observed in the low pass condition, F(1, 34) = 14.22, p<.01, η^2^_p_ = .30.

Finally, a significant interaction was observed between Filter and Step, indicating that the P1 amplitude was more positive for low-pass than for high-passed stimuli in the initial identification steps, and did not differ in the four final steps, F(15, 510) = 2.2, p<.01, η^2^_p_ = .06. Overall, a significantly more positive P1 amplitude was observed for low-passed than for high-passed pictures, F(1, 34) = 35.27, p<.001, η^2^_p_ = .51.

### N1

Waveforms for the N1 component are reported in Figure 2 (lateral sites). The amplitude of N1 was more negative in the first steps than in the later steps, F(15, 510) = 4.94, p<.01, η^2^_p_ = .13. A significant interaction between Step and Filter was observed, F(15, 510) = 3.61, p<.001, η^2^_p_ = .10, indicating that the effect of Step was more pronounced in the low-pass than in the high-pass condition. Following this significant interaction, we observed that the effect of Step on the amplitude of the N1 was significant in the low-pass condition, F(15, 510) = 8.13, p<.001, η^2^_p_ = .19, but only approached standard significance in the high-pass condition, F(15, 510) = 2.1, p = .076, η^2^_p_ = .06.

### P2

**Figure 3:**
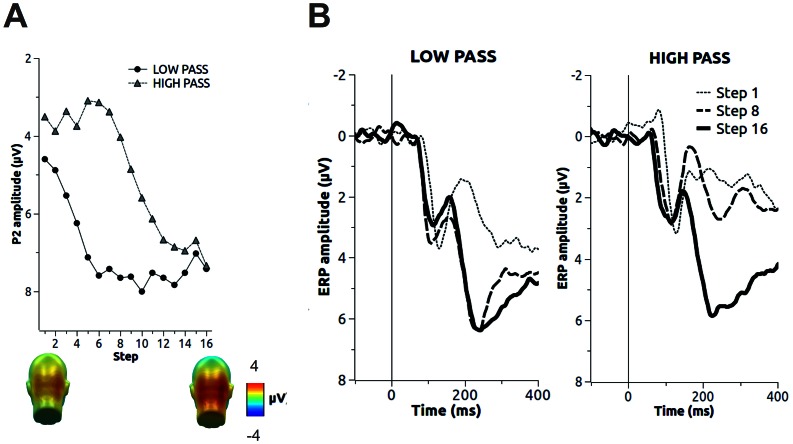
The effects of spatial frequencies on the P2. A. The modulation of the P2 by filter step. The topographies corresponding to the first and last step of the procedure are displayed at the bottom of the line plot, averaged for low- and high-passed pictures. B. Waveforms of the first, intermediate and final step of the procedure, in the low-pass and high-pass condition. Waveforms represent the average across sensors in central and lateral sensor groups. In all plots, positive values are plotted downwards.

The effects of spatial frequencies on P2 amplitude are reported in Figure 3. The amplitude of the P2 was more positively pronounced in the final than in the initial steps, F(15, 510) = 22.32, p<.001, η^2^_p_ = .40. A significant interaction between Step and Filter was observed, F(15, 510) = 16.3, p<.001, η^2^_p_ = .32, indicating that low-passed pictures elicited a more pronounced P2 compared to that produced for high-pass filters in steps 1 to 13. No significant difference between low-passed and high-passed pictures was observed in the three final steps. Overall, the amplitude of the P2 was more positive for low-passed than for high-passed pictures, F(1, 34) = 119.94, p<.001, η^2^_p_ = .78.

### Cortical Sources

Based on the above findings, further analyses were restricted to the P1 and P2 component, as these components showed the strongest effects of Filter Type and Cutoff. The cortical sources for the grand averaged ERPs were estimated using sLORETA. The descriptive results of this analysis are shown in Figure 2 and in Table 1. Briefly, the cortical generators of the midline P1 that was observed in the first steps were localized in the striate cortex (Brodmann areas 17 and 18). The generators of the lateral P1, as well as of the P2, were localized in the middle occipital cortex (Brodmann area 19).

**Table 1:** MNI coordinates of the maximum estimated sLORETA activity values corresponding to the P1 and P2 ERP components.

ERP	Condition	Brodmann area	X	Y	Z
P1	First Step	Left cuneus, Brodmann area 17	−5	−100	−5
	First Step	Right cuneus, Brodmann area 17	5	−101	−6
	First Step	Left middle occipital gyrus,Brodmann area 18	−19	−101	5
	First Step	Right middle occipital gyrus,Brodmann area 18	20	−101	5
	Last Step	Left middle occipital gyrus,Brodmann area 19	−40	−90	3
	Last Step	Right middle occipital gyrus,Brodmann area 19	36	−90	4
P2	First Step	Left middle occipital gyrus,Brodmann area 19	−39	−90	6
	First Step	Right middle occipital gyrus,Brodmann area 19	35	−90	5
	Last Step	Left middle occipital gyrus,Brodmann area 19	−40	−90	5
	Last Step	Right middle occipital gyrus,Brodmann area 19	40	−90	3

### Regression Analyses

**Figure 4:**
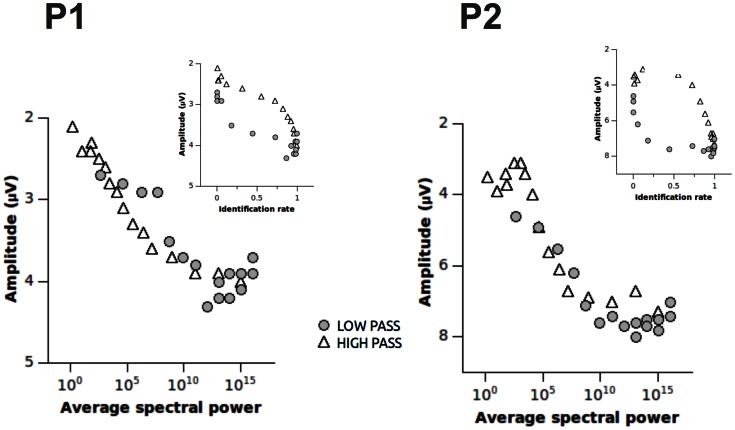
The relationship between spectral power, identification and the amplitude of the P1 (left) and of the P2 (right). In the main panels, the relationship between the spectral power and ERP amplitude is displayed, separately for low- and high-passed pictures. In the insets, the relationship between identification rate and ERP amplitude is represented.

The relationship between ERP amplitude and spectral power, picture identification and spatial frequencies is shown in Figure 4. A linear relationship was observed between the amplitude of the P1 and P2, and the averaged spectral power. Finally, ERP amplitude increased with identification rate, but the exact relationship between identification and ERP amplitude depended on the frequency range of the picture.

To substantiate these findings, and to disentangle the role of spectral power, identification and spatial frequency on the amplitude of the P1 and of the P2, a stepwise multiple regression was conducted. The results of this analysis are reported in Table 2. As far as the P1 is concerned, a model including only spectral power explained 86% of the total variance. Additionally, the inclusion of identification rate and spatial frequencies explained an additional 6% of variance. Regarding the P2, a model including spectral power alone explained 83% of the total variance, and adding identification rate and spatial frequencies did not significantly increase the variance accounted for by the model.

**Table 2:** Summary of regression analysis for variables predicting the amplitude of the P1 and P2.

P1									
	Model 1			Model 2			Model 3		
Variable	B	SE B	β	B	SE B	β	B	SE B	β
Spectral Power	.12	.009	.925	.086	.012	.663	.053	.019	.412
Identification rate				.539	.142	.341	.826	.190	.522
Spatial Frequency							.257	.121	.199
R^2^	.86			.90			.92		
*F* for change in *R^2^*	176.8***			14.37**			4.5*		
**P2**									
	**Model 1**								
**Variable**	**B**	**SE B**	**β**						
Spectral Power	.305	.025	.912						
Identification rate									
Spatial Frequency									
R^2^	.83								
*F* for change in *R^2^*	148.61***								

B: unstandardized beta coefficient; SE B: standard error of B. β: standardized beta coefficient.

* *p*<.05.

** *p*<.01.

*** *p*<.001.

## Discussion

The present study examined the identification of natural scenes based on low or high spatial frequency information. The results pointed out a more pronounced role for spectral power, than for low spatial frequencies and scene identification, in the modulation of the extrastriate activity reflected in the lateral P1 and in the P2.

Previous studies investigating the identification of fragmented objects showed effects of stimulus identification on the P2, with more pronounced amplitude for intact compared to degraded objects [11]. Here this effect was replicated, both for low-pass and high-pass filtered natural scenes. However, the effect was more pronounced for low spatial frequencies, and the regression analysis showed that picture identification had no role in P2 modulation, once spectral power was controlled for. Degraded objects differ from intact objects in that they comprise a smaller contrast across spatial frequencies and, overall, a smaller spectral power [3]. This result is consistent with several studies, which have demonstrated the sensitivity of early ERPs to sensory and perceptual parameters such as contrast and salience [27], [28].

It is a well-replicated result that a family of occipital positive ERPs peaking about 200 ms from stimulus onset are modulated by factors such as target status [29]–[31], attention [32], and motivational relevance [33] which are not closely bound to low-level features. Additionally, these components are sensitive to grouping and closure phenomena [7], [8], [28], [34]–[36], and the P2 has been observed to be modulated by perceptual implicit memory [37]. Taking these findings together, the P2 appears to index an intermediate processing stage that is closely bound to perceptual processes such as segmentation or grouping and higher-level processes such as categorization.

The close link between perceptual segmentation and object categorization is supported by several studies investigating the categorization of degraded objects and the related neural correlates [38], [39]. Here, the dominant generators of the P2 were localized in the lateral occipital extrastriate areas. The lateral occipital complex (LOC) is functionally defined as the brain region in which metabolic activity is enhanced for intact compared to scrambled objects [40], [41]. As such, the LOC responds more strongly to coherent figures than to meaningless visual input [38], [42]. Similarly, activity in this region is sensitive to categorization [43] and to the relationship between objects [44]. In our study, the amplitude of the P2 was clearly related to the degradation of natural scenes, suggesting a functional similarity between the modulation of this component and the activity of the LOC. Altogether, the present results support the link between the P2 and the activity on the LOC in terms of source localization and of sensitivity to scene degradation.

Two temporally separated stages of processing which were sensitive to scene degradation were observed, corresponding to the P1 and to the P2. Both ERPs were localized in similar regions for intact stimuli, and quite likely reflected separate processing stages, carried out to analyze features of increasing complexity. The lateral P1 and the P2 showed similar functional properties, as they were both modulated by the amount of contrast contained in the visual input. However, these components differed in the topography and with respect to their neural generators. The activity reflected by the P2 was localized in the extrastriate visual areas, regardless of the complexity of the visual input. On the other hand, the P1 reflected striate activation for highly degraded stimuli, and extrastriate activation for more complex scenes.

At the initial identification steps, a clear P1 peak was observed, which was generated by activity in the striate visual cortex. While classic P1 studies report a lateral topography for this component, as well as extrastriate generators [45], the results of a recent study may suggest that the midline peak observed here may be related to striate processing of background noise. Electrocortical responses to backgrounds and foregrounds flickering at different frequencies were examined. Activity in the LOC was only observed for figures, while backgrounds resulted in activation of the striate areas [46]. In the present results the midline P1 was localized in the striate cortex and was only observed for the most degraded pictures, while more lateral activity was observed for less degraded stimuli. It is possible that, following the presentation of highly degraded stimuli, brain areas which are related to the segregation and categorization of meaningful stimuli are not recruited, and processing is only carried out in primary striate areas. However, due to important methodological differences between the present and the cited study, further research is necessary to investigate this possibility.

In the present study, we observed that the amplitude of the lateral P1 and of the P2 increased for high- and low-passed scenes similarly once spectral power was controlled for. In natural contexts, the frequency power of the visual input usually decreases with increasing spatial frequencies [16]. In the present study the filtering of natural images produced picture versions where some ranges of frequencies were missing, and the spectral power of the stimuli used here hence varied with the type and cutoff of the filter. A clear linear relationship was observed between the spectral power of the images and the amplitude of the extrastriate activity, regardless of the type of filtering that was applied (low- or high-pass). This result suggests that extrastriate activity reflected in the lateral P1 and in the P2 is sensitive to the spectral power of the visual input rather than tuned to a specific range of spatial frequencies.

Previous studies which examined the effects of spatial frequencies on ERPs using gratings and checkerboard stimuli demonstrated a more negative C1 for high-frequency stimuli, and a more positive P1 for low-pass stimuli [7], [9]. Here, these effects were replicated and extended to natural pictures. An interesting finding was that, using complex pictures, the effects of high spatial frequencies on the C1 amplitude appeared to be less pronounced compared to previous studies using gratings and checkerboards. A recent study examined the modulation of C1 by stimuli of increasing complexity, and reported a notable C1 to high-frequency gratings that was progressively dampened as stimulus complexity increased [7]. One possibility is that natural scenes, being composed of a wide range of spatial frequencies, are not such potent elicitors of the C1 as high-frequency gratings are. Alternatively, as the polarity of the C1 is known to be reversed for stimuli presented in the upper and lower visual hemifield, it may be that natural scenes extending in both the upper and lower hemifield may have reduced the amplitude of the C1 [47]. Future studies might further investigate the effects of spatial frequencies on C1 amplitude, focusing on the differences between natural scenes and simpler stimuli and on the visual hemifield where scenes are presented.

### Limitations and Future Directions

In the present study scene identification was examined in a setting in which initially degraded stimuli were progressively revealed. Due to the sequential nature of this paradigm, it is very likely that participants developed hypotheses as to the identity of the visual stimulus, which were matched against the available perceptual evidence [48]. Top-down processes are a fundamental part of visual perception, as they bias the processing of visual scenes based on previous knowledge about the stimuli, the attentional setting and the current aims [4]. Moreover, top-down influences modulate the amplitude of the P2 [49] and the activity in the LOC [50], [51]. Therefore, it is important that future studies examine the contribution of top-down factors such as attentional setting and task at hand in the modulation of the P2 by picture degradation.

A methodological implication of the present data concerns studies which aim to examine the role of spatial frequencies in emotional response, focusing on early cortical processes [52]. Here it is shown that, both for pictures consisting of low and those consisting of high spatial frequencies, the total spectral power modulates the activity of the extrastriate areas, with more positive amplitude of the scalp P2 amplitudes for stimuli with a high spectral power. Therefore it may be important to match pictures on this physical dimension, as well as other low level properties, in order to examine early emotion-related ERP effects. Moreover, it should be noted that emotional effects are most consistently observed and investigated on a component, namely the Late Positive Potential (LPP), which is observed in a later time window and with a different topography compared to the ERP components which were studied in the present study [53], [54].

This study investigated the role of low and high spatial frequencies on the visual processing stages which eventually lead to scene identification, and revealed effects which built up as scenes of increasing completeness were presented. As noted above, picture completeness covaried with spectral power and identification, and the multiple linear regression helped us to disentangle the role of scene identification and of spectral power. In particular, a more important role for spectral power in modulating the amplitude of the extrastriate activity was observed, compared to low spatial frequencies and scene identification. However, to substantiate the present findings, future studies could compare the neural processing of visual information which is matched for spectral power but differs in meaningfulness, for instance by comparing natural scenes to meaningless stimuli of matched complexity, such as phase-scrambled pictures [55], [56]. Moreover, alternative measures of identification may be used, such as asking participants to name images or to decide whether pictures match a target category or template [19], [57].

### Conclusion

Here, we investigated the neural correlates of the early visual processing of natural scenes. The amplitude of the P1 and P2 were modulated as pictures became less degraded, probably reflecting the progressive engagement of extrastriate areas such as the lateral occipital complex. No effects were observed that were specific to the spatial frequency range, and the degradation-related effects were linearly related to the spectral power of natural scenes.

## Supporting Information

Figure S1 **Sensor groups used for analysis.** Configuration of the 257 sensors of the EEG cap. Sensors used for the ERP analysis are colored in gray.
(TIFF)
