# Supplementary figures and images for: Early Spatial Frequency Processing of Natural Images: An ERP Study
Source: PLoS One. 2013 May 31;8(5):e65103. doi: 10.1371/journal.pone.0065103 (PMC3669057; doi:10.1371/journal.pone.0065103)

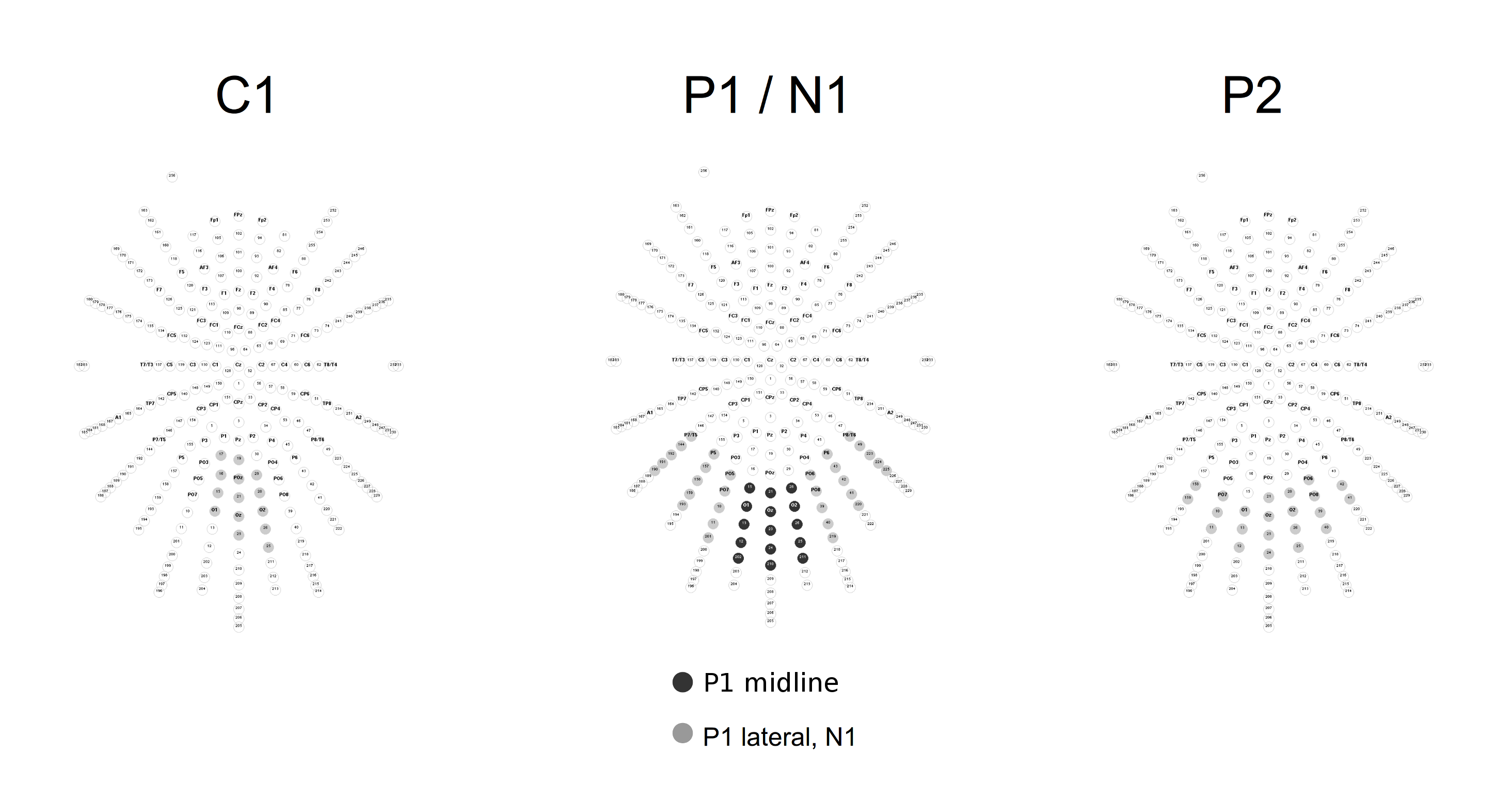

Supplement: Figure S1 — Sensor groups used for analysis. Configuration of the 257 sensors of the EEG cap. Sensors used for the ERP analysis are colored in gray. (TIFF) [file pone.0065103.s001.tiff]
